# Dynamic regulation and function of histone monoubiquitination in plants

**DOI:** 10.3389/fpls.2014.00083

**Published:** 2014-03-13

**Authors:** Jing Feng, Wen-Hui Shen

**Affiliations:** Institut de Biologie Moléculaire des Plantes, UPR2357 CNRS, Université de StrasbourgStrasbourg, France

**Keywords:** chromatin, epigenetics, ubiquitin, histone monoubiquitination, transcription regulation, plant development, *Arabidopsis thaliana*

## Abstract

Polyubiquitin chain deposition on a target protein frequently leads to proteasome-mediated degradation whereas monoubiquitination modifies target protein property and function independent of proteolysis. Histone monoubiquitination occurs in chromatin and is in nowadays recognized as one critical type of epigenetic marks in eukaryotes. While H2A monoubiquitination (H2Aub1) is generally associated with transcription repression mediated by the Polycomb pathway, H2Bub1 is involved in transcription activation. H2Aub1 and H2Bub1 levels are dynamically regulated *via* deposition and removal by specific enzymes. We review knows and unknowns of dynamic regulation of H2Aub1 and H2Bub1 deposition and removal in plants and highlight the underlying crucial functions in gene transcription, cell proliferation/differentiation, and plant growth and development. We also discuss crosstalks existing between H2Aub1 or H2Bub1 and different histone methylations for an ample mechanistic understanding.

## INTRODUCTION

Ubiquitin (Ub) and Ub-like (e.g., SUMO) proteins constitute a family of modifiers that are linked covalently to target proteins. Although ubiquitination (also called ubiquitylation or ubiquitinylation) first came to light in the context of protein destruction, it is now clear that ubiquitination can also carry out proteolysis-independent functions. Ubiquitination can alter biochemical, molecular and/or subcellular localization activities of a target protein. The first ubiquitinated protein to be described was histone H2A in calf thymus, a finding dated more than 36 years ago (Goldknopf et al., 1975; Hunt and Dayhoff, 1977). Yet, only more recently have the underlying mechanisms and regulatory functions of histone ubiquitination begun to emerge (reviewed in Zhang, 2003; Shilatifard, 2006; Weake and Workman, 2008; Braun and Madhani, 2012; Pinder et al., 2013). Histones are highly alkaline proteins, found in the nuclei of eukaryotic cell, which package and order the DNA into structural units named nucleosomes. A nucleosome is composed of roughly 146 bp of DNA wrapping around the histone octamer comprising two molecules each of the four core histones H2A, H2B, H3, and H4 (Luger et al., 1997). Histone monoubiquitination together with other types of posttranslational modifications, e.g., acetylation, methylation, phosphorylation, and SUMOylation, can modulate nucleosome/chromatin structure and DNA accessibility and thus regulate diverse DNA-dependent processes, such as genome replication, repair, and transcription (Zhang, 2003; Shilatifard, 2006; Weake and Workman, 2008; Braun and Madhani, 2012; Pinder et al., 2013).

Ubiquitination occurs *via* conjugation of the C-terminal residue of Ub to the side chain of a lysine (K) residue of the substrate/acceptor protein, a reaction involving three coordinated enzymatic activities (reviewed in Hershko and Ciechanover, 1998). Ub is first activated by an ATP-dependent reaction involving the Ub-activating enzyme E1, then conjugated to the active site cysteine residue of the Ub-conjugating (UBC) enzyme E2, and finally transferred to the target K residue of the substrate protein by the Ub-protein isopeptide ligase E3. Most organisms have only one E1, but dozens of different E2 and hundreds up to thousands of different E3 enzymes, providing the need in coping with effective substrate specificity (Hua and Vierstra, 2011; Braun and Madhani, 2012). Identification and characterization of E3s and some E2s involved in histone ubiquitination had been a key for understanding biological functions of histone ubiquitination in various organisms. Because of its suitability for genomics, genetics, and cellular and molecular biological approaches, *Arabidopsis thaliana* is an ideal model to investigate histone ubiquitination functions. In this review, we focus on this reference plant to expose current progress made on ubiquitination of different types of histones.

## H2B MONOUBIQUITINATION IN *Arabidopsis*

### GENOME-WIDE DISTRIBUTION OF H2Bub1

Monoubiquitinated H2B (H2Bub1) was first discovered in mouse cells and was estimated to represent about 1–2% of total cellular H2B (West and Bonner, 1980). Later, H2Bub1 was detected widely throughout eukaryotes spanning from yeast to humans and plants (Zhang, 2003; Shilatifard, 2006; Sridhar et al., 2007; Zhang et al., 2007a; Weake and Workman, 2008). The ubiquitination site is mapped to a highly conserved K residue, H2BK123 in budding yeast, H2BK119 in fission yeast, H2BK120 in humans, and H2BK143 in *Arabidopsis*. Genome-wide analysis revealed that in *Arabidopsis* as in animals H2Bub1 is associated with active genes distributed throughout the genome and marks chromatin regions notably in combination with histone H3 trimethylated on K4 (H3K4me3) and/or with H3K36me3 (Roudier et al., 2011). During early photomorphogenesis, gene upregulation was found to be associated with H2Bub1 enrichment whereas gene downregulation did not show detectable correlation with any H2Bub1 level changes (Bourbousse et al., 2012). In general, H2Bub1 is considered to represent an active chromatin mark broadly involved in genome transcription regulation.

### ENZYMES INVOLVED IN REGULATION OF H2Bub1 LEVELS

The budding yeast Rad6 (radiation sensitivity protein 6) was the first factor identified and shown to work as an E2 enzyme involved in catalyzing H2Bub1 formation both *in vitro* and *in vivo* (Robzyk et al., 2000). It contains a highly conserved catalytic UBC domain of approximately 150 amino acids in length with an active-site cysteine for linking Ub. The E3 enzyme working together with Rad6 in catalyzing H2Bub1 formation in budding yeast is Bre1 (Brefeldin-A sensitivity protein 1), which contains a C3HC4-type RING finger domain typical for all E3s (Hwang et al., 2003; Wood et al., 2003). The depletion of either Rad6 or Bre1 eliminates genome-wide H2Bub1 and causes yeast cell growth defects (Robzyk et al., 2000; Hwang et al., 2003; Wood et al., 2003). Human contains at least two homologs of Rad6, namely hHR6A and hHR6B, and two homologs of Bre1, namely RNF20/hBRE1A and RNF40/hBRE1B (Kim et al., 2005; Zhu et al., 2005). In *Arabidopsis*, three homologs of Rad6, namely UBC1, UBC2, and UBC3, were identified and UBC1 and UBC2 but not UBC3 were shown to be redundantly responsible for H2Bub1 formation *in planta *(Cao et al., 2008; Gu et al., 2009; Xu et al., 2009). The two Bre1 homologs HUB1 (HISTONE MONOUBIQUITINATION 1) and HUB2 work non-redundantly, possibly as a hetero-tetramer composed of two copies of HUB1 and two copies of HUB2, in catalyzing H2Bub1 formation in *Arabidopsis* (Fleury et al., 2007; Liu et al., 2007; Cao et al., 2008). H2Bub1 levels are drastically reduced or undetectable in Western blot analysis in the loss-of-function* hub1 *and *hub2* single mutants as well as in the *hub1 hub2* and *ubc1 ubc2* double mutants, but are unaffected in the *ubc1*, *ubc2, *and *ubc3 *single mutants or in the* ubc1 ubc3* and *ubc2 ubc3 *double mutants (Cao et al., 2008; Gu et al., 2009; Xu et al., 2009).

H2Bub1 levels are also regulated by deubiquitination enzymes. Two Ub-specific proteases, Ubp8 and Ubp10, are involved in deubiquitination of H2Bub1 in budding yeast. Strikingly, while Ubp8 acts as a component of the SAGA (Spt-Ada-Gcn5-acetyltransferase) complex specifically in H2Bub1 deubiquitination in transcription activation, Ubp10 functions independently of SAGA and primarily acts in Sir-mediated silencing of telomeric and rDNA regions (reviewed in Weake and Workman, 2008). In human, USP22 acts as Ubp8 ortholog in a SAGA complex in H2Bub1 deubiquitination (Weake and Workman, 2008). In *Arabidopsis*, although a SAGA complex remains uncharacterized so far, the Ub protease UBP26/SUP32 has been shown to deubiquitinate H2Bub1 involved in both heterochromatic silencing (Sridhar et al., 2007) and transcription activation of the *FLC* (*FLOWERING* LOCUS C) gene (Schmitz et al., 2009). More recently, the otubain-like deubiquitinase OTLD1 was reported as implicated in deubiquitination of H2BUb1 and repression of *At5g39160*, a gene of unknown function (Krichevsky et al., 2011).

### ROLE OF H2Bub1 IN FLOWERING TIME REGULATION

**FIGURE 1:**
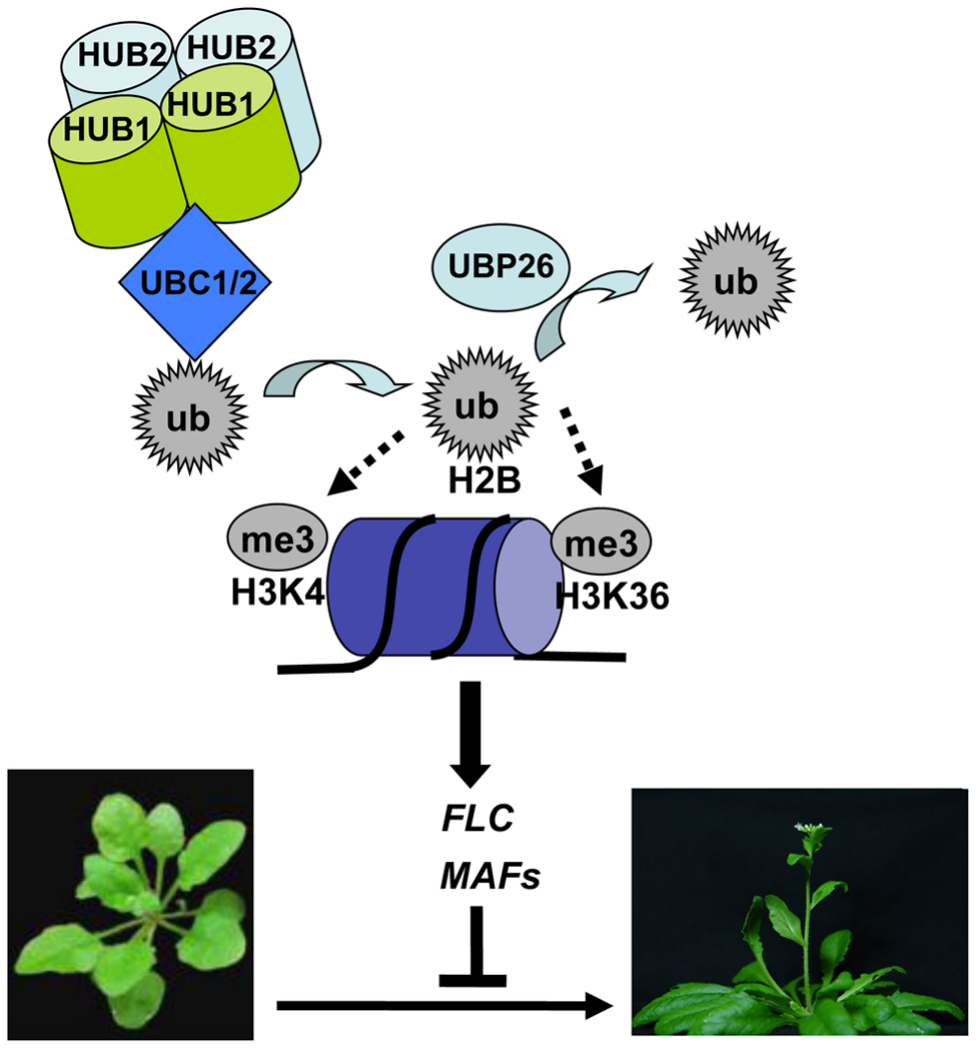
**A proposed model for deposition and removal of histone H2B monoubiquitination in transcriptional activation of *FLC* and *MAFs* in flowering time regulation.** In this model, HUB1 and HUB2 form a heterotetramer and recruit UBC1 or UBC2 to *FLC*/*MAFs* chromatin, leading to transfer of a ubiquitin (ub) monomer from UBC1 or UBC2 onto H2B. H2Bub1 formation enhances H3K4me3 deposition by methyltransferases, together promoting transcription initiation. UBP26 removes ubiquitin on H2B, favoring H3K36me3 deposition in promoting transcription elongation. Active transcription of *FLC/MAFs* represses *Arabidopsis *flowering, a transition from vegetative to reproductive plant development.

The timing of flowering is critical for the reproductive success of plants. As compared to wild type, the* hub1 *and *hub2* single mutants as well as the *hub1 hub2* and *ubc1 ubc2* double mutants exhibit an early flowering phenotype whereas but the *ubc1*, *ubc2, *and *ubc3 *single mutants and the* ubc1 ubc3* and *ubc2 ubc3 *double mutants have a normal phenotype (Cao et al., 2008; Gu et al., 2009; Xu et al., 2009). This early flowering phenotype is detectable under both long-day and short-day photoperiod plant growth conditions. Molecular analyses of the mutants indicate that H2Bub1 controls flowering time primarily through transcriptional activation of *FLC* (**Figure 1**). *FLC* encodes a key transcription repressor involved in both the autonomous/developmental and vernalization flowering pathways, and its active transcription is associated with several histone marks, e.g., H3K4me3, H3K36me2/3 and H2Bub1 (reviewed in Berr et al., 2011). In the early flowering mutants *hub1*, *hub2*, *hub1 hub2,* and *ubc1 ubc2*, *FLC* expression levels are reduced and the *FLC* chromatin shows reduced H2Bub1 levels (Cao et al., 2008; Gu et al., 2009). The loss-of-function mutant *ubp26*/*sup32* showed also an early flowering phenotype and reduced *FLC* expression but an elevated level of H2Bub1 in the *FLC* chromatin (Schmitz et al., 2009), indicating that not only H2Bub1 formation but also H2Bub1 removal are necessary for *FLC* transcription. Accompanying H2Bub1 reduction compromised levels of H3K4me3 and to a less extent H3K36me2 were detected at *FLC* in *hub1* and *ubc1 ubc2* (Cao et al., 2008), and reduced level of H3K36me3 but elevated level of H3K27me3 was observed at *FLC* in *ubp26/sup32* (Schmitz et al., 2009). On parallels to the knowledge in yeast, it was proposed that the UBC-HUB-mediated H2Bub1 formation is necessary for H3K4me3 deposition at transcription initiation whereas UBP26/SUP32-mediated H2Bub1 removal is required for H3K36me3 deposition during transcription elongation (Cao et al., 2008; Schmitz et al., 2009). Nonetheless, this hierarchy of histone modifications needs to be cautioned because multiple factors are involved in H3K4me3 and H3K36me2/3 depositions and the SDG8 (SET DOMAIN GROUP 8)-mediated H3K36me2/3 deposition remarkably override H3K4me2/3 deposition in *FLC* transcription (Yao and Shen, 2011; Shafiq et al., 2014). Besides *FLC*, *Arabidopsis* has five *FLC* paralogs, namely *MAF1* (*MADS AFFECTING FLOWERING 1*), *MAF2,* *MAF3*, *MAF4* and *MAF5*. Some *MAFs* are also downregulated in the early flowering mutants *hub1*, *hub2*, *hub1 hub2*, *ubc1 ubc2, *and* ubp26/sup32 *(Cao et al., 2008; Gu et al., 2009; Schmitz et al., 2009; Xu et al., 2009). Thus, H2Bub1 may also regulate flowering time through control of *MAF* gene expression under some plant growth conditions.

### H2Bub1 FUNCTION IN OTHER PROCESSES

In addition to flowering, many other processes also involve H2Bub1 as evidenced by studies of the *Arabidopsis* *hub1* and *hub2* mutants. The *hub *mutants display reduced seed dormancy associated with reduced expression of several dormancy-related genes, including *DOG1 *(*DELAY OF GERMINATION 1*), *ATS2 *(*ACYLTRANSFERASE 2*), *NCED9 *(*NINE-CIS-EPOXYCAROTENOID DIOXYGENASE 9*), *PER1 *(*CYSTEINE PEROXIREDOXIN 1*), and* CYP707A2 *(Liu et al., 2007). At vegetative growth stages, the *hub* mutants exhibit pale leaf coloration, modified leaf shape, reduced rosette biomass, and inhibited root growth (Fleury et al., 2007). Cell cycle genes, particularly some key regulators of the G2-to-M transition, are downregulated, which could largely explain the plant growth defects of the *hub* mutants (Fleury et al., 2007). A more recent study shows that several circadian clock genes, including *CCA1 *(*CIRCADIAN CLOCK ASSOCIATED 1*), *ELF4 *(*EARLY FLOWERING 4*) and *TOC1 *(*TIMING OF CAB EXPRESSION 1*), are downregulated and their chromatin regions contain lower levels of H2Bub1 in the *hub* mutants, suggesting that H2Bub1 may contribute to the regulation of plant growth fitness to environment through expression modulation of some circadian clock genes (Himanen et al., 2012). It is worth to note that SDG2-mediated H3K4me3 deposition is also required for expression of several circadian clock genes (e.g., *CCA1, TOC1*) and the *hub* mutants exhibit reduced levels of H3K4me3 in chromatin regions of the circadian clock genes (Himanen et al., 2012; Malapeira et al., 2012).

During photomorphogenesis, hundreds of genes show upregulation associated with H2Bub1 enrichment in their chromatin in response to light exposure (Bourbousse et al., 2012). Strikingly, over 50% of these genes gain H2Bub1 enrichment upon the 1 h of illumination, illustrating the highly dynamic nature of H2Bub1 deposition during a likely cell division-independent genome regrogramming process. In contrast to the above discussed cases, in this study the H2Bub1 changes is neither accompanied by any detectable changes of H3K36me3 nor required for H3K4me3 enrichment following six hours of light exposure (Bourbousse et al., 2012). In line with the function of H2Bub1 in gene activation in response to light, the *hub1-3* mutant seedlings are overly light sensitive, exhibiting a photobleaching phenotype (Bourbousse et al., 2012).

The *hub1* mutants also show increased susceptibility to the necrotrophic fungal pathogens *Botrytis cinerea* and *Alternaria brassicicola* (Dhawan et al., 2009). Precise role of H2Bub1 in plant defense against pathogens still remains largely unclear. Structure defects, e.g., thinner cell walls and altered surface cutin and wax compositions, together with impaired induction of some defense genes might have partly contributed to the increased susceptibility to pathogen infection in the *hub* mutant plants (Dhawan et al., 2009; Ménard et al., 2014). It is worthy noting that the *sdg8* mutants impaired in H3K36me3 deposition also display reduced resistance to necrotrophic fungal pathogen infection (Berr et al., 2010, 2012; Palma et al., 2010). It will be interesting to study in future research whether a trans-histone crosstalk between H2Bub1 and H3K36me3 acts on transcription induction in plant response to pathogens.

### MECHANISMS OF H2Bub1 IN TRANSCRIPTION REGULATION

So far only limited information is available concerning how H2Bub1 enzymes are recruited to the target chromatin. The evolutionarily conserved PAF1 (Polymerase Associated Factor 1) complex interacts with Pol II (RNA polymerase II) and plays a role as a “platform” for association of enzymes involved in H2bub1, H3K4me3, and H3K36me2/3 deposition, linking histone modifications with active transcription (Shilatifard, 2006; Weake and Workman, 2008; Berr et al., 2011; Braun and Madhani, 2012). A direct interaction between PAF1 complex and Rad6-Bre1 has been detected and shown as required for catalyzing H2Bub1 formation (Xiao et al., 2005). As in yeast and animals, deletion or knockdown of PAF1 components markedly reduces H2Bub1 in *Arabidopsis* (Schmitz et al., 2009). Genetic analysis shows that *HUB2* and *ELF8* encoding a PAF1 subunit act in a same floral-repression pathway in *Arabidopsis* flowering time regulation (Gu et al., 2009). Although physical interaction between UBC-HUB and PAF1 needs future investigation, interactions were observed between UBC and HUB (Cao et al., 2008) and between HUB and MED21 (mediator complex subunit 21), a subunit of the evolutionarily conserved Mediator complex (Dhawan et al., 2009). Mediator complex is associated with both general transcription factors and Pol II and is essential for activator-dependent transcription in all eukaryotes (for a recent review, see Carlsten et al., 2013). Nevertheless, the aforementioned interactors are generally involved in Pol II transcribed genes and thus cannot fully explain why UBC-HUB targets some but not all active genes. It is reasonable to speculate that UBC-HUB recruitment might also involve some gene-specific yet uncharacterized factors.

The next question is how H2Bub1 affects transcription. In yeast and animals, H2Bub1 can promote transcription elongation by enhancing the recruitment of RNA Pol II and by facilitating nucleosome removal through interplay with FACT (facilitates chromatin transcription), an evolutionarily conserved histone chaperone complex (Pavri et al., 2006; Tanny et al., 2007). FACT acts on displacement of H2A/H2B dimer from a nucleosome core, facilitating transcription elongation on chromatin template. In *Arabidopsis*, FACT genetically interacts with HUB1 and plays critical roles in multiple plant developmental processes (Lolas et al., 2010). Yet its precise interplay with H2Bub1 in transcription regulation needs future investigations.

Alternatively or additionally, H2Bub1 may regulate transcription indirectly through crosstalk with H3K4me3 and H3K36me2/3 (Shilatifard, 2006; Weake and Workman, 2008; Berr et al., 2011; Braun and Madhani, 2012). In line with this idea, lack of H2Bub1 in *Arabidopsis* impairs H3K4me3 and H3K36me2 formation in chromatin at *FLC* and clock genes (Cao et al., 2008; Himanen et al., 2012), and elevated H2Bub1 inhibits H3K36me3 formation in the *FLC* chromatin (Schmitz et al., 2009). Nevertheless, in contrast to the requirement of H2Bub1 for genome-wide H3K4me3 formation in yeast, lack of H2Bub1 in *Arabidopsis* barely affects global H3K4me2/3 and H3K36me2/3 levels, as evidenced by Western blot analysis (Cao et al., 2008; Dhawan et al., 2009; Gu et al., 2009) as well as by ChIP (chromatin immunoprecipitation) analysis of light responsive genes during photomorphogenesis (Bourbousse et al., 2012). It is currently unclear to which extent applies the crosstalk of H2Bub1 with H3K4me2/3 and H3K36me2/3 in *Arabidopsis* gene transcription regulation and what are the molecular mechanisms underlying the crosstalk.

Finally, while H2Bub1 is generally associated with active gene transcription, it can also regulate transcription repression in a chromatin context-dependent manner. The *ubp26/sup32* mutant shows release of transgene and transposon silencing (Sridhar et al., 2007) as well as elevated expression of *PHE1* (*PHERES1*) associated with seed developmental defects (Luo et al., 2008). It has been shown that the silencing release is accompanied by reduction of H3K9me2 and of siRNA-mediated DNA methylation and the *PHE1* expression elevation is associated with a reduced level of H3K27me3. Nevertheless, whether these changes of repressive marks are directly linked with H2Bub1 still need to be investigated.

## H2A MONOUBIQUITINATION IN *Arabidopsis*

### PRESENCE OF H2Aub1

In contrast to H2Bub1, H2Aub1 has not been found in yeast and has been generally implicated in transcription repression in animal cells (Weake and Workman, 2008; Braun and Madhani, 2012). Albeit its early discovery and high abundance (about 5–15% of the total H2A) in animal cells (Goldknopf et al., 1975; Hunt and Dayhoff, 1977; Zhang, 2003), H2Aub1 function has only more recently begun to be elucidated, thanking to the first identification of the human PRC1 (Polycomb repressive complex 1) component Ring1B (also known as Ring2 and RNF2) as a E3 involved in catalyzing H2Aub1 formation (Wang et al., 2004). In *Arabidopsis*, H2Aub1 was undetectable in a large-scale analysis of histone post-translational modifications by mass spectrometry (Sridhar et al., 2007; Zhang et al., 2007a) and had been thought for a long time to be non-existent (Weake and Workman, 2008). However, five PRC1-like RING-finger proteins, namely AtRING1a, AtRING1b, AtBMI1a, AtBMI1b, and AtBMI1c, have been identified in *Arabidopsis* (Sanchez-Pulido et al., 2008; Xu and Shen, 2008). More recent immunodetection and *in vitro* enzyme activity assays have revealed that these RING-finger proteins are effectively involved in catalyzing H2Aub1 formation in *Arabidopsis* (Bratzel et al., 2010; Li et al., 2011; Yang et al., 2013).

### PRC2 AND PRC1 IN H2Aub1 DEPOSITION

Polycomb group (PcG) proteins, first identified in *Drosophila* as repressors of homeotic (*Hox*) genes, are nowadays known to act in multiprotein complexes in transcription repression of a large number of genes in many multicellular organisms including plants (Bemer and Grossniklaus, 2012; Molitor and Shen, 2013; Schwartz and Pirrotta, 2013; Simon and Kingston, 2013). The most intensively studied complexes are PRC1 and PRC2. In *Drosophila*, PRC2 is composed of four core subunits, namely Ez (Enhancer of zeste), Suz12 (Suppressor of zeste 12), Esc (Extra sex combs) and N55 (a 55 kDa WD40 repeat protein), and PRC1 also contains four main subunits, namely Pc (Polycomb), Ph (Polyhomeotic), Psc (Posterior sex combs) and Ring1 (also known as dRing). In mammals, alternate subunit compositions create larger families of related PRC2-type and PRC1-type complexes (Schwartz and Pirrotta, 2013; Simon and Kingston, 2013). Nevertheless, defined biochemical activities of PRC2 and PRC1 are conserved from flies to humans. The classical model proposes a sequential mode of action of the two complexes: PRC2 catalyzes H3K27me3 formation, and PRC1 recognizes the H3K27me3 mark and further mediates downstream H2Aub1 deposition. The PRC1 components, acting as E3 ligases in H2Aub1 formation, are RING-finger proteins: Ring1 in *Drosophila* and Ring1A and Ring1B in human (Braun and Madhani, 2012; Schwartz and Pirrotta, 2013).

**FIGURE 2:**
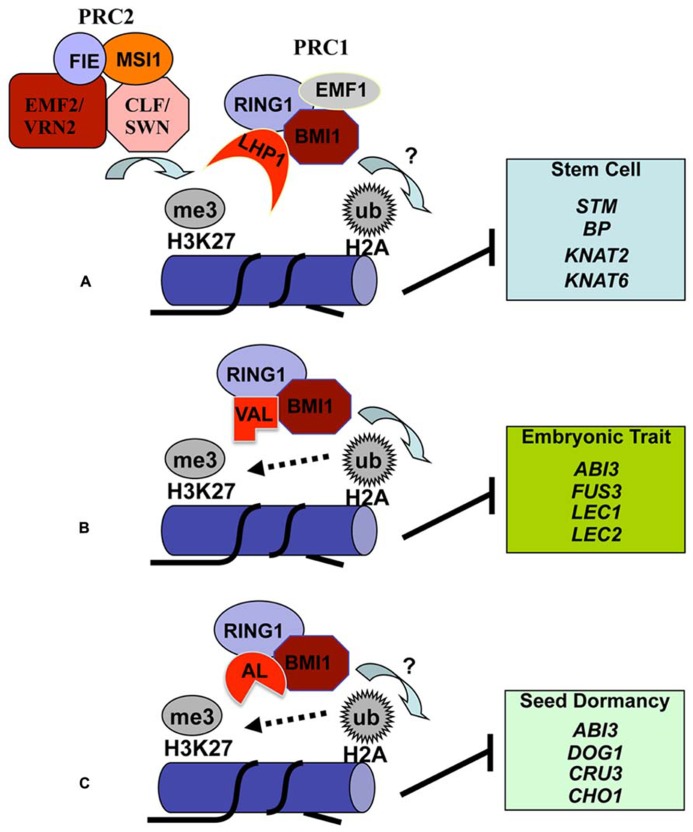
**Proposed models for histone H2A monoubiquitination deposition in transcriptional repression of varied target genes.** The *Arabidopsis *PRC1-like RING-finger proteins AtRING1a/b (RING1) and AtBMI1a/b/c (BMI1) have the E3 ligase activity in catalyzing H2A monoubiquitination (H2Aub1). Comparable to the classical model of sequential PRC2 then PRC1 action in Polycomb silencing in animal cells, the *Arabidopsis *PRC1-like protein LHP1 binds H3K27me3 pre-deposited by the evolutionarily conserved PRC2 complexes and recruits RING1, BMI1 and possibly also EMF1 through protein–protein interactions **(A)**. This combinatorial action by PRC2 then PRC1 likely plays a broad role in suppression of numerous genes, including the key stem cell regulatory *KNOX* genes that need to be stably repressed during lateral organ development. The transcription factor VAL is involved in recruitment of BMI1 and RING1 in suppression of embryonic trait genes in somatic cells **(B)**. AL proteins bind BMI1 and RING1 and play important roles in suppression of several key seed dormancy regulatory genes to promote germination **(C)**. H3K27me3 deposition at embryonic/seed genes is enhanced by VAL/AL-PRC1 **(B,C)**, unraveling a non-canonical crosstalk between H3K27me3 and H2Aub1. The question marks indicate that H2Aub1 deposition in the specified target gene chromatin still requires future investigation.

In *Arabidopsis*, the four PRC2 core components are highly conserved (**Figure 2**) and encoded by small gene families, and their function in H3K27me3 deposition and transcription repression have been intensively studied (Bemer and Grossniklaus, 2012). In contrast, PRC1 compositions are drastically diverged in plants as compared to animals (Molitor and Shen, 2013). No sequence homologue of Ph could be identified in**plants so far. LHP1 (LIKE HETEROCHROMATIN PROTEIN 1), also known as TFL2 (TERMINAL FLOWER 2), binds H3K27me3 and may play a Pc-like function (Turck et al., 2007; Zhang et al., 2007b). This remarkably differs from the distinct roles of HP1 and Pc in animals, where HP1 binds H3K9me3 involved in hetereochromatin formation whereas Pc binds H3K27me3 involved in PRC1-mediated silencing in euchromatin. The best conservations found about PRC1 core components are from RING-finger proteins structured by a RING domain at N-terminus and a Ub-like RAWUL domain at C-terminus (Sanchez-Pulido et al., 2008; Xu and Shen, 2008). These RING-finger proteins can be classified into two phylogenic groups: the first group comprises *Drosophila* Ring1, human Ring1A and Ring1B, and *Arabidopsis* AtRING1a and AtRING1b; the second group comprises *Drosophila* Psc, human Bmi1, and *Arabidopsis* AtBMI1a, AtBMI1b, and AtBMI1c. Consistent with their sequence conservation, AtRING1a, AtRING1b, AtBMI1a, and AtBMI1b each can ubiquitinate H2A *in vitro*, and loss of function of *AtBMI1a* and *AtBMI1b* causes H2Aub1 reduction *in planta* (Bratzel et al., 2010; Yang et al., 2013).

### ROLE OF PRC1-LIKE RING-FINGER PROTEINS IN STEM CELL MAINTENANCE

Plant growth and development largely depend on stem cells located in SAM (shoot apical meristem) and RAM (root apical meristem), whose activities are fine-tuned by multiple families of chromatin factors (Sang et al., 2009; Shen and Xu, 2009). The first uncovered biological role of the *Arabidopsis* PRC1-like RING-finger proteins are on the regulation of SAM activity (Xu and Shen, 2008). While the single loss-of-function mutants *Atring1a *and *Atring1b* have a normal phenotype, the double mutant *Atring1a Atring1b* exhibits enlarged SAM, fasciated stem, and ectopic-meristem formation in cotyledons and leaves. This indicates that *AtRING1a* and *AtRING1b* play a redundant role in stable repression of stem cell activity to allow appropriate lateral organ differentiation. The balances between stem cell maintenance and cell differentiation for organ formation are controlled by specific transcription factors, including KNOX (Class I KNOTTED1-like homeobox) proteins. Strikingly, several *KNOX* genes, e.g., *STM *(*SHOOT-MERISTEMLESS*), *BP* (*BREVIPEDICELLUS*)/*KNAT1*, *KNAT2* and *KNAT6,* are upregulated in *Atring1a Atring1b *(Xu and Shen, 2008). Ectopic expression of *KNOX* genes colocalizes with and precedes ectopic meristem formation. It has been proposed that AtRING1a/b acts as a crucial PRC1 component in conjunction with PRC2 in repression of *KNOX* genes to promote lateral organ formation in the SAM (**Figure 2A**).

### ROLE OF PRC1-LIKE RING-FINGER PROTEINS IN EMBRYONIC CELL FATE DETERMINACY

Further characterization of the ectopic meristem structures observed in *Atring1a Atring1b* unravels that these callus structures exhibit embryonic traits (Chen et al., 2010). The *Atbmi1a Atbmi1b* mutant also displays derepression of embryonic traits (Bratzel et al., 2010; Chen et al., 2010). Embryonic callus formation has been observed broadly in somatic tissues of cotyledons, leaves, shoots and roots of the mutant plants. Treatment with an auxin transport inhibitor can inhibit embryonic callus formation in *Atring1a Atring1b*, indicating that a normal auxin gradient is required for somatic embryo formation in the mutant (Chen et al., 2010). Both *Atring1a Atring1b *and *Atbmi1a Atbmi1b* mutants exhibit elevated expression of several key embryonic regulatory genes, including *ABI3 *(*ABSCISIC ACID INSENSITIVE 3*)*, AGL15 *(*AGAMOUS LIKE 15*)*, BBM *(*BABYBOOM*), *FUS3 *(*FUSCA 3*)*, LEC1 *(*LEAFY COTYLEDON 1*), and* LEC2* (Bratzel et al., 2010; Chen et al., 2010). It is likely that derepression of these regulatory genes together with *KNOX* has contributed to the ectopic meristem and embryonic callus formation in somatic tissues of the *Atring1a Atring1b *and *Atbmi1a Atbmi1b* mutants (**Figure 2B**). The VAL (VP1*/*ABI3*-*LIKE) transcription factors can physically interact with AtBMI1 proteins and the *val1 val2 *mutant exhibits comparable phenotype to *Atbmi1a Atbmi1b*, suggesting that VAL and AtBMI1 proteins may form complexes in repression of embryonic regulatory genes during vegetative development (Yang et al., 2013). Notably, loss of VAL or AtBMI1 causes H2Aub1 reduction in chromatin regions at *ABI3*, *BBM*, *FUS3* and *LEC1* but not *STM* (Yang et al., 2013). Future investigation is necessary to clarify whether AtBMI1 and AtRING1 proteins repress *KNOX* transcription *via* H2Aub1 deposition or other independent chromatin remodeling mechanisms.

### ROLE OF PRC1-LIKE RING-FINGER PROTEINS IN SEED GERMINATION

Seed germination defines the entry into a new generation of the plant life cycle. It is generally accepted that the process of germination starts with water uptake followed by seed coat rupture and is completed following radicle protrusion (Bentsink and Koornneef, 2008). During the very early phase, the embryonic growth program remains latent and can be reinstated in response to unfavorable environmental cues. With the attainment of photosynthetic competence, the irreversible transition to autotrophic growth is accomplished and embryonic program is stably suppressed. A recent study (Molitor et al., 2014) has identified the *Arabidopsis* PHD-domain H3K4me3-binding AL (ALFIN1-like) proteins as interactors of AtBMI1 and AtRING1 proteins and has demonstrated a crucial function of chromatin state switch in establishment of seed developmental gene repression during seed germination (**Figure 2C**). Loss of AL6 and AL7 as well as loss of AtBMI1a and AtBMI1b retards seed germination and causes transcriptional derepression and a delayed chromatin state switch from H3K4me3 to H3K27me3 enrichment of seed developmental genes, including *ABI3* and *DOG1*. The germination delay phenotype of the *al6 al7* and *Atbmi1a Atbmi1b* mutants is more pronounced under osmotic stress (Molitor et al., 2014), suggesting that AL PHD-PRC1 complexes may participate in regulation of seed germination in response to environmental cues.

### ROLE OF PRC1-LIKE RING-FINGER PROTEINS IN OTHER PROCESSES

AtBMI1a and AtBMI1b, also named DRIP1 (DREB2A-INTERACTING PROTEIN 1) and DRIP2, had been reported first as E3 ligases involved in ubiquitination of DREB2A (DEHYDRATION-RESPONSIVE ELEMENT BINDING PROTEIN 2A), a transcription factor controlling water deficit-inducible gene expression (Qin et al., 2008). The *drip1 drip2* mutant shows enhanced expression of water deficit-inducible genes and more tolerance to drought (Qin et al., 2008). Overexpression of *AtBMI1c* accelerates flowering time, which is associated with reduction of *FLC *expression**(Li et al., 2011). In addition to SAM maintenance defects and derepression of embryonic traits, the *Atring1a Atring1b* mutant also displays homeotic conversions of floral tissues (Xu and Shen, 2008). Therefore, more precise functions and underlying molecular mechanisms for the PRC1-like RING-finger proteins are still waiting to be uncovered during plant development and in plant response to environmental changes.

### MECHANISMS OF PRC1-LIKE RING-FINGER PROTEINS IN TRANSCRIPTION REPRESSION

H2Aub1 function in plants is primarily evidenced through investigation of roles of the *Arabidopsis* PRC1-like RING-finger proteins (Xu and Shen, 2008; Bratzel et al., 2010; Chen et al., 2010; Li et al., 2011; Yang et al., 2013). Although these RING-finger proteins act nicely *in vitro* as E3 ligases, their *in vivo* functions in H2Aub1 deposition are still poorly documented. H2Aub1 level in *Arabidopsis* seems very low because large-scale analyses of either the histone-enriched or the Ub-affinity-purified protein preparations fail to detect H2Aub1 (Maor et al., 2007; Sridhar et al., 2007; Zhang et al., 2007a; Manzano et al., 2008; Saracco et al., 2009). H2Aub1 has been detected only by using specific antibodies, and in this case *AtBMI1* genes have been shown to act as positive regulators for H2Aub1 deposition in *Arabidopsis* plants (Bratzel et al., 2010; Li et al., 2011; Yang et al., 2013). It is unknown whether any deubiquitinases might cause low levels of H2Aub1 in *Arabidopsis*. In animal cells, several deubiquitinases are characterized as specific for H2Aub1 (Weake and Workman, 2008; Simon and Kingston, 2013). Future characterization of *Arabidopsis* H2Aub1 deubiquitinases may provide useful information regarding regulatory mechanisms of H2Aub1 dynamics.

AtRING1 and AtBMI1 proteins physically interact each other and with the H3K27me3-binding protein LHP1 (Xu and Shen, 2008; Bratzel et al., 2010; Chen et al., 2010), providing a possible recruitment mechanism similar to the classical sequential PRC2 then PRC1 silencing pathway in animal cells. However, the *Atring1a Atring1b*, *Atbmi1a Atbmi1b,* or *Atbmi1a Atbmi1b Atbmi1c* mutant exhibits much more severe phenotypic defects than the *lhp1* mutant does, and *lhp1* enhances the *Atring1a Atring1b* mutant defects. It is thus apparent that AtRING1 and AtBMI1 proteins also act independently from LHP1. Recent identification of the transcriptional regulator VAL as AtBMI1-binding protein and of AL as AtRING1 and AtBMI1 interactor provides some novel insight about recruitment mechanisms (Yang et al., 2013; Molitor et al., 2014). It is particular intriguing that loss of AtBMI1 impairs H3K27me3 enrichment at seed developmental genes during seed germination and vegetative growth (Yang et al., 2013; Molitor et al., 2014). It has also been reported that loss of LHP1 impairs H3K27me3 enrichment at flower gene loci in roots (Derkacheva et al., 2013). These recent findings challenge the classic hierarchical paradigm where PRC2-mediated H3K27me3 deposition precedes PRC1 recruitment (**Figure 2**). It is obvious that future investigations are necessary to better understand the composition and function of different PRC1-like complexes in *Arabidopsis*.

## CONCLUSIONS AND PERSPECTIVES

Studies over the last few years in the model plant *Arabidopsis* have greatly advanced our knowledge about the roles of H2Aub1 and H2Bub1 in transcription regulation in plant growth and development. In view of additional functions described in animal cells for both H2Aub1 and H2Bub1 in DNA damage repair (Bergink et al., 2006; Marteijn et al., 2009; Chernikova et al., 2010; Ginjala et al., 2011; Moyal et al., 2011; Nakamura et al., 2011), it is anticipated that more roles of H2Aub1 and H2Bub1 in plant response to environmental stresses are waiting to be uncovered. Mutagenesis of enzymes involved in H2Aub1 and H2Bub1 deposition or removal is required to address the question whether these enzymes effectively exert their biological functions *via* H2Aub1 and H2Bub1. Identification and characterization of factors associated with these different enzymes will be essential for understanding molecular mechanisms of their recruitment and function at specific targets within the genome. We need to know whether and how their function is spatially and temporally integrated with plant development. Genome-wide tools need to be further explored to provide a global view of links among enzyme or associated factor binding, H2Aub1/H2Bub1 enrichment, H3 methylation, and Pol II occupation. Crosstalks between H2Aub1 or H2Bub1 and different H3 methylations need to be addressed for chromatin context specificity.

In addition to H2Aub1 and H2Bub1, ubiquitinated H1, H3, and H4 are also found in *Arabidopsis* (Maor et al., 2007; Manzano et al., 2008; Saracco et al., 2009). H3 ubiquitination catalyzed by Rtt101-Mms1 in yeast and by Cul4-DDB1 in human has been recently shown to play an important role in the histone chaperone Asf1-mediated nucleosome assembly (Han et al., 2013). *Arabidopsis* contains a conserved family of CULLINs and CUL4-DDB1 complexes are reported (Shen et al., 2002; Hua and Vierstra, 2011). The Asf1 homologues in *Arabidopsis* are also identified (Zhu et al., 2011). It remains to be investigated whether CUL4-DDB and AtASF1 collaboratively act on nucleosome assembly *via *H3 ubiquitination in epigenetic regulation in *Arabidopsis*.

## Conflict of Interest Statement

The authors declare that the research was conducted in the absence of any commercial or financial relationships that could be construed as a potential conflict of interest.
